# Porous Geopolymer/ZnTiO_3_/TiO_2_ Composite for Adsorption and Photocatalytic Degradation of Methylene Blue Dye

**DOI:** 10.3390/polym15122697

**Published:** 2023-06-15

**Authors:** Ximena Jaramillo-Fierro, Sneyder Gaona, John Ramón, Eduardo Valarezo

**Affiliations:** 1 Departamento de Química, Facultad de Ciencias Exactas y Naturales, Universidad Técnica Particular de Loja, San Cayetano Alto, Loja 1101608, Ecuador; bevalarezo@utpl.edu.ec; 2 Ingeniería Química, Facultad de Ciencias Exactas y Naturales, Universidad Técnica Particular de Loja, San Cayetano Alto, Loja 1101608, Ecuador; sagaona1@utpl.edu.ec (S.G.); jbramon2@utpl.edu.ec (J.R.)

**Keywords:** geopolymer, ZnTiO_3_/TiO_2_, metakaolin, adsorption, photocatalysis, methylene blue

## Abstract

In this study, GP (geopolymer) and GTA (geopolymer/ZnTiO_3_/TiO_2_) geopolymeric materials were prepared from metakaolin (MK) and characterized by X-ray diffraction (XRD), X-ray fluorescence (XRF), scanning electron microscopy (SEM), energy dispersive X-rays (EDX), specific surface area (SSA), and point of zero charge (PZC). The adsorption capacity and photocatalytic activity of the compounds prepared in the form of pellets was determined by degradation of the methylene blue (MB) dye in batch reactors, at pH = 7.0 ± 0.2 and room temperature (20 °C). The results indicate that both compounds are highly efficient at adsorbing MB, with an average efficiency value of 98.5%. The Langmuir isotherm model and the pseudo second order kinetic model provided the best fits to the experimental data for both compounds. In the MB photodegradation experiments under UVB irradiation, GTA reached an efficiency of 93%, being higher than that achieved by GP (4%). Therefore, the incorporation of ZnTiO_3_/TiO_2_ in the geopolymeric matrix allowed GTA to achieve higher overall efficiency, by combining adsorption and photocatalysis, compared to the GP compound. The results indicate that the synthesized compounds could be used for up to five consecutive cycles for the removal of MB from wastewater through adsorption and/or photocatalysis processes.

## 1. Introduction

Modern industrial development has resulted in many benefits derived from socioeconomic development, but it has also resulted in environmental pollution and adverse health impacts. These impacts are relevant when considering industries with large production volumes [1]. Industries release large amounts of water with a variety of chemicals [2]. One of these chemicals is dye, of which approximately 700,000 tons are produced [3] and 280,000 tons are discharged into effluents annually [4]. The textile industry is the largest consumer of dyes, with a consumption of 10,000 different types of colorants [3]. One of these dyes is methylene blue (MB) [5]. MB has various applications in paper, food, pharmaceuticals, and textile industries. In the textile industry, MB is used to color cotton, wool, and silk, among other materials, because of its resistance to water, soap and oxidizing agents [6]. The MB discharged into effluents does not allow the penetration of sunlight, which is a way to reduce photosynthetic action [7]. In addition to environmental damage, it has also been shown that effluents contaminated by dyes can affect human health. When inhaled in large quantities, MB can cause painful urination and respiratory complications, and when ingested, it can cause burning, nausea, vomiting, and mental confusion [8].

The dyes have complex degradation pathways; biological, chemical and physicochemical processes have all been implemented as decontamination techniques for dyes [9]. Among the physicochemical treatments, coagulation, filtration, ionic exchange and adsorption can be mentioned. Among the chemical treatments are ozonation and oxidation. In biological treatments there is activated sludge, pure cultures (fungi, bacteria) and mixed cultures (anaerobic and aerobic decomposition) [10]. Currently, within the oxidation processes, those known as Advanced Oxidation Processes (AOPs) have been developed [11]. AOP have advantages over other conventional treatments, such as the transformation of organic compounds into simple inorganic compounds (H_2_O, CO_2_ and salts) allowing their removal in another stage of treatment; they can also be useful in pre-treatment so that the recalcitrant pollutants (compounds with a complex structure such as hydrocarbons, drugs) can be biologically treated before being discharged into the environment [12]. Heterogeneous photocatalysis is a process that is part of the AOPs and this treatment is characterized by the use of UV (visible ultraviolet) light, that through semiconductor catalysts allow the transformation of organic compounds to inorganic compounds. Photocatalysis refers to a catalytic reaction that involves the absorption of light by a catalyst or substrate. Both light and catalyst are necessary to achieve or speed up a chemical reaction. Thus, photocatalysis can be defined as the acceleration of a photoreaction by means of a catalyst [13]. The initial stage of the process consists of the generation of an electron-hole pair in the semiconductor particles. When a photon with energy that equals or exceeds the bandgap energy of the semiconductor hits it, an electron is promoted from the valence band to the conduction band, generating a hole in the valence band. The bandgap energy is the energy difference of the valence band and the conduction band of a photocatalyst [14]. Among the semiconductors used in this process we have cadmium sulfide (CdS), titanium oxide (TiO_2_), zinc oxide (ZnO) and zinc sulfide (ZnS) [15].

Titanium oxide is a semiconductor allotrope that has become one of the most widely used in photocatalysis. Its applications are environmental due to factors such as its chemical stability, its photoactivity, low operating costs and low toxicity [16]. It is made up of three crystalline phases: anatase, brookite and rutile. Anatase has an easy preparation and photocatalytic activity [17]. Anatase has a bandgap of 3.2 eV, while the rutile is 3.0 eV, but many investigations have focused on anatase for its high photocatalytic efficiency, negligible toxicity and its low cost [18]. In addition to TiO_2_, titanium is also found in other photocatalytic compounds, such as compounds from the perovskite family. A perovskite is any material that has the same type of crystalline structure as calcium titanate (CaTiO_3_), for example zinc titanate (ZnTiO_3_) [19]. Zinc titanate is not part of TiO_2_ and ZnO semiconductors, on the contrary, it is formed by these, which have a broad band which cannot be activated with visible light. ZnTiO_3_ is characterized by its thermal and chemical stability, the energy is wide in its forbidden band with a value of 2.9 eV to 3.59 eV, this allows it to be photoactive in the presence of UV light, allowing high electron mobility [20]. ZnTiO_3_ is very cheap, has almost negligible environmental impact [21], and is used as a catalytic adsorbent (gas desulfurization), bacterial agent, gas sensor and photocatalyst [22]. ZnTiO_3_ can improve its photocatalytic efficiency if it is doped with metals or through heterojunction with other semiconductors such as TiO_2_ to form a ZnTiO_3_/TiO_2_ hybrid semiconductor [23].

In order to improve the mechanical properties of photocatalyst materials and reduce the amount of material used, some research has focused on using different supports, such as cementitious materials, clays, and geopolymers for photocatalysts [24]. Geopolymers are inorganic polymers formed by aluminum and silicon tetrahedral units. Their structure consists of a Si–O–Al polymer framework, with corners that share oxygen, forming a 3D network with metal cations as well as water stabilizing the structure [25]. The geopolymers are obtained by geopolymerization, which is a chemical reaction between the source of aluminosilicates and the alkaline solution commonly composed of sodium hydroxide and sodium silicate [26]. Furthermore, geopolymerization is a process in which small molecules come together to form an amorphous 3D network [26]. Geopolymers present properties such as low CO_2_ emission in their production, high adsorption capacity, high chemical stability, good compressive strength, high durability, mechanical resistance, and thermal stability, due to their amorphous polymeric structures. Although most of the applications of geopolymers are oriented to the construction field, they can also be used as supports for photocatalysts [27]. The materials that are used in the production of geopolymers regardless of the application must contain a high level of silica and alumina, which make up approximately 65% of the earth’s crust. There are several types of precursors, such as fly ash, kaolin, zeolites, rice hulls, etc. Of these, kaolin has a higher silicon and aluminum content, making it more suitable as a raw material for the synthesis of geopolymers [28]. Kaolin is a white non-reactive material that is composed of hydrated aluminum silicate, with a 1:1 structural ratio. This is formed by two layers, in the first it has tetrahedrons that allow the silicon atoms to concentrate in the center and the oxygens in their vertices, the second layer is formed by octahedrons with central aluminum atoms and hydroxyl groups with oxygen atoms [29]. This type of mineral is considered dioctahedral, occupied by aluminum, this is like trioctahedral minerals, belonging to magnesium and iron.

Dense geopolymers are heavy with low machinability. Porous geopolymers have advantages, such as a nanoscale size, large volume pores and large specific surface area, compare with dense geopolymers [30]. The advantage of geopolymers compared to raw clays and other classic adsorbents is their high mechanical resistance, chemical and thermal stability, allowing their regeneration and recycling [31]. Among the methods to obtain porous polymers are self-forming, direct foaming, adding filler and particles stacking methods [32]. Pure porous geopolymer in different forms (granules, monoliths, etc.) have been used as adsorbents in aqueous systems for volatile organic compounds (VOC), for heavy metals and other contaminants such as ammonium ions and dyes, in gaseous systems for CO_2_ removal, and for immobilization of toxic and radioactive waste [33]. The advantage of using geopolymers in the adsorption process is their easy regeneration and reuse after exhaustion in the production of new construction materials. Within the wastewater treatment of the textile industry, porous geopolymeric materials are viable alternatives for the removal of MB [34]. Porous geopolymer composites can be applied in membrane support, water purification, thermal insulation materials and lightweight parts. Porous geopolymer composites with high mechanical strength are used in building, sorbent and catalyst materials [35]. In catalysis geopolymers are used as support for catalytic or photocatalytic materials [36].

In previous studies it has been determined that porous geopolymers can be used to remove MB dye from aqueous solutions and wastewaters [31,32,33,34,37], however, the efficiency of dye removal can be increased by working with porous geopolymer composites filled with a semiconductor that helps photodegradation of the dye [30]. In this context, in a recent study it was shown that the photocatalyst ZnTiO_3_/TiO_2_ is an efficient compound for the removal of MB dye [38]. However, the ZnTiO_3_/TiO_2_ compound needs to be supported in a matrix that provides a distribution surface to reduce the amount of compound used and which also provides the possibility of the conformation in manageable materials, such as pellets and extrusions. The fact that these extrusions can be easily removed once the photocatalysis has been carried out, and that a porous geopolymer has not been used as a support for the ZnTiO_3_/TiO_2_ photocatalyst compound, has motivated us to carry out the present study with the aim of synthesizing a porous geopolymer from kaolin, characterize it, and use it as a support for ZnTiO_3_/TiO_2_, and apply the resulting material to the removal of MB from aqueous systems.

## 2. Materials and Methods

**Figure 1:**
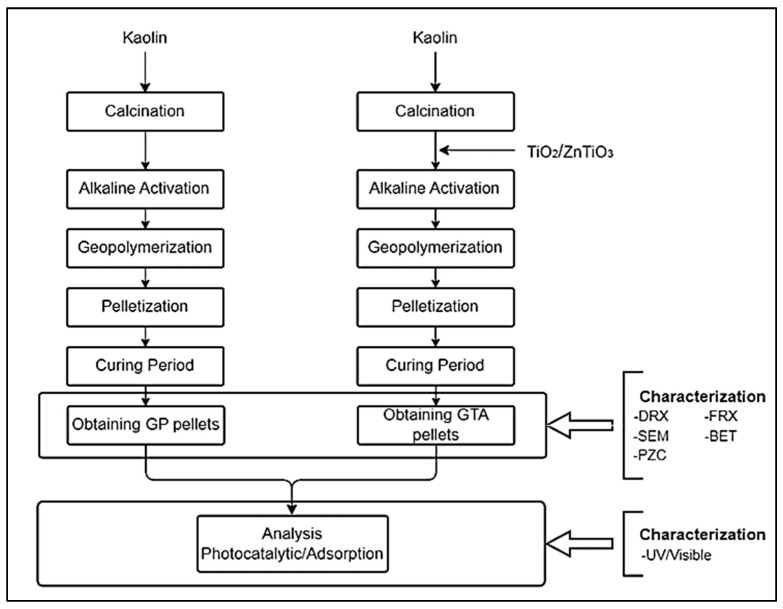
Schematic representation of the research method.

The scheme in Figure 1 provides an overview of the methodology used and helps to understand the logical sequence of the steps involved in this study.

### 2.1. Materials

All chemicals (analytical grade) were utilized in this research without additional purification: titanium (IV) isopropoxide (Ti(OC_3_H_7_)_4_, Sigma Aldrich, St. Louis, MO, USA, 98.0%), zinc acetate ((CH_3_CO_2_)_2_Zn, Sigma Aldrich, 99.99%), isopropyl alcohol (C_3_H_8_O, Sigma Aldrich, ≥99.5%), hydrochloric acid (HCl, Sigma Aldrich, 37.0%), sodium hydroxide (NaOH, Sigma Aldrich, ≥85.0%), hydrogen peroxide (H_2_O_2_, Sigma Aldrich, 35%), sodium metasilicate nonahydrate (Na_2_O_3_Si·9H_2_O, Sigma Aldrich, ≥98.0%), kaolin (Sigma Aldrich), sodium dodecyl sulfate (CH_3_(CH_2_)_11_OSO_3_Na, Sigma Aldrich, ≥99.0%), poly(ethylene glycol) (H(OCH_2_CH_2_)_n_OH, Sigma Aldrich).

### 2.2. Synthesis of GP and GTA Compounds

The geopolymeric compounds were synthesized following a modified method described by other authors [39]. To prepare the geopolymeric compound called GP (geopolymer only) firstly, kaolin was calcined at 650 °C for 3 h to dehydrate it (dehydroxylation) and obtain metakaolin (MK). The alkaline activator was prepared by incorporating 1.67 g of NaOH to 8.83 g of sodium silicate solution and 2.76 mL of distilled water. A quantity of 10 g of metakaolin was incorporated into the activating solution and sonicated until complete homogenization. Then, 0.39 g of sodium dodecyl sulfate and 0.27 mL of hydrogen peroxide were added, and sonication was carried out again until a homogeneous paste was obtained. With this paste, cylindrical pellets with dimensions of 1.0 cm long and 0.25 cm in diameter were manufactured. These pellets were prepared using a 2.5 cm diameter syringe and were allowed to settle for approximately 1 h. After this time, the pellets were suspended in a polyethylene glycol (PEG) bath at a temperature of 85 ± 5 °C to allow their complete solidification. During this process, the fully submerged geopolymer pellets in the bath were evenly covered by the PEG, which acts as a support and confinement medium for the geopolymer, providing a controlled environment for the solidification and curing reaction. The PEG bath must be kept at a suitable temperature (85 ± 5 °C) to promote the polymerization and crosslinking reaction of the geopolymer. Contact with the PEG bath helps to accelerate the solidification and curing reaction of the geopolymer, improving its mechanical resistance and dimensional stability. PEG also acts as a heat transfer medium, allowing for more precise temperature control during the solidification process. Once the required solidification time in the PEG bath was completed, the geopolymer pellets were removed from the bath and further cured at 45 °C and 65% humidity for 24 h to complete formation of the geopolymeric structure. During curing, additional reactions occurred that improved the properties of the geopolymer, such as its strength, durability, and chemical stability.

To obtain the geopolymeric compound called GTA (geopolymer/ZnTiO_3_/TiO_2_), the methodology explained above was used, but with a 3:7 ratio of ZnTiO_3_/TiO_2_:MK instead of the pristine metakaolin (MK). The combination of the ZnTiO_3_/TiO_2_ hybrid semiconductor with the metakaolin was carried out by mechanical mixing [40]. The synthesis of ZnTiO_3_/TiO_2_ nanoparticles was carried out following the sol-gel method described in previous investigations [38]. To obtain the ZnTiO_3_/TiO_2_ hybrid semiconductor, a solution of titanium (IV) isopropoxide (TIPP) and isopropyl alcohol (iPrOH) at a TIPP/iPrOH ratio of 70% *v/v* was prepared at room temperature. At the same time, a solution of zinc acetate dihydrate, isopropyl alcohol and water was prepared, to achieve a 3:1 TiO_2_/ZnO molar ratio. The iPrOH/water (50% *v/v*) ratio was established by stoichiometry, to allow hydrolysis of titanium isopropoxide. The titanium isopropoxide solution was slowly added to the zinc acetate solution while maintaining constant stirring at room temperature. As a result of this process, a white precipitate was obtained, which was dried at 60 °C for 24 h and then calcined at 500 °C for 4 h. After the curing process, the GP and GTA pellets were characterized and evaluated regarding their ability for adsorption and photodegradation of MB in aqueous solutions.

### 2.3. Characterization

This investigation utilized various methods to analyze the samples. X-ray diffraction (XRD) measurements were performed by means of a Bruker-AXS D8-Discover diffractometer (Bruker AXS, Karlsruhe, Germany), which produced Cu Kα radiation with a wavelength of 1.5406 Å. Data acquisition took place in the angular range of 5 to 90° in terms of 2θ. The Crystallography Open Database (COD database, version 2021) was used to recognize the crystalline phases. X-Ray fluorescence (XRF) analysis was conducted by means of a Bruker S1 Turbo SDR portable spectrometer (Bruker Handheld LLC, Kennewick, WA, USA). This analysis utilized the Mining Light Elements measurement method. To measure the specific surface area (SSA) of the samples, nitrogen adsorption was at a temperature of −196 °C. The adsorption process was performed using a gas mixture consisting of 30% nitrogen (N_2_) diluted in helium (He) in the ChemiSorb 2720 equipment (Micromeritics, Norcross, GA, USA). The SSA was estimated applying the Brunauer-Emmet-Teller (BET) equation with the Chemisoft TPx system (version 1.03; data analysis software; Micromeritics, Norcross, GA, USA, 2011) by the single-point method. Micrographs and energy-dispersive X-ray (EDX) spectra were obtained employing a JEOL JSM 6400 scanning electron microscope (SEM-EDX) (JEOL, Peabody, MA, USA). The photoactivity of the samples was evaluated at λ = 310 nm using the IPW-UV-610 Stainless Steel Inner Sterilizer Light (IPW Industries Inc., Santa Ana, CA, USA), and the amount of MB residual in the solutions was quantified at λ = 490 nm using a Jenway 7350 spectrophotometer (Cole-Parmer, Staffordshire, UK). The study also involved determining the point of zero charge (PZC) for all the samples at room temperature (20 ± 2 °C) by applying the pH drift method, where ΔpH = pH_f_ − pH_i_ = 0. The experiment was carried out by adding 0.1 g of the solid sample to a 50 mL tube. In this tube, there was 25 mL of a 0.1 M NaCl solution. The pH of the solutions was adjusted to values ranging from 3 to 10 using 0.1 M HCl or NaOH solutions, and these values were noted as pH_i_. The tubes were then agitated for 24 h at 250 rpm, and the final pH of the supernatant liquid in each tube was measured and noted as pH_f_. By plotting ΔpH (ΔpH = pH_f_ − pH_i_) vs. pH_i_, the PZC was determined. This process was repeated for both geopolymeric compounds using 0.01 and 0.05 M NaCl solutions. The tests were repeated three times, and the average value of the point of zero charge (pH_PZC_) was calculated and reported for each sample [41].

### 2.4. Adsorption Studies

In this study, MB dye adsorption assays from aqueous solutions were considered to assess the effect of solution pH, initial dye concentration, reaction temperature, and adsorbent-adsorbate contact time. The data accomplished from these experiments were fitted to isotherm and kinetic models by the least squares nonlinear regression method [42]. The assays were carried out in batch systems at room temperature using the methodology reported in previous studies [43]. The pH of the solutions was corrected to 7.0 ± 0.2 by adding 0.1 M solutions of sodium hydroxide (NaOH) or hydrochloric acid (HCl). A constant amount of 200 mg L^−1^ of geopolymeric compounds was assessed in all assays. To determine the maximum adsorption capacity of MB, the concentration of 500 mL of the dye solution was varied from 0.5 to 50 mg L^−1^. The effect of pH, temperature and contact time on the adsorption of MB on the materials was carried out by utilizing a 500 mL volume of water that contained a dye concentration of 20 mg L^−1^. The residual MB concentration in the solution was verified by UV–vis spectrophotometry at 623 nm, based on the previously prepared calibration curve (R^2^ = 0.9995) according to the Lambert–Beer Law. The assays were conducted in triplicate, and the results were reported as the average of the three repetitions [43].

The following equation was employed to estimate the amount of dye (q_e_) that was adsorbed on the geopolymeric compounds [44]:

(1) $$q_{e}=\left(C_{0}-C_{e}\right)\times \frac{v}{w}$$

where C_0_ and C_e_ denote the initial and equilibrium concentrations of MB dye, respectively, and are evaluated in mg L^−1^. The mass of the adsorbent (w) and the volume of the solution (v) are expressed in grams (g) and liters (L), respectively.

To evaluate the equilibrium MB adsorption, the Langmuir and Freundlich isotherm models were utilized. The Langmuir isotherm model is expressed by the subsequent equation [45]:

(2) $$\frac{C_{e}}{q_{e}}=\frac{1}{K_{L}q_{\max}}+\frac{C_{e}}{q_{\max}}$$

where q_max_ in mg g^−1^ represents the maximum monolayer adsorption. The Langmuir constant K_L_ (L mg^−1^) is associated with the adsorption energy at equilibrium. C_e_ in mg L^−1^ represents the concentration of solute at equilibrium. In addition, the R_L_ separation factor can be evaluated using the following equation to provide information about the adsorption characteristics [45]:

(3) $$R_{L}=\frac{1}{\left(1+K_{L}C_{0}\right)}$$

The R_L_ factor is useful to estimate the suitability of the adsorption process, thus: if R_L_ = 1 the adsorption is linear, if R_L_ > 1 the adsorption is unfavorable, if R_L_ = 0 the adsorption is irreversible, and if 0 < R_L_ < 1 the adsorption is favorable.

On the other hand, the Freundlich isotherm model is expressed by the subsequent equation [45]:

(4) qe=KFCe1n

where the Freundlich constant (K_F_) expressed in L mg^−1^ represents the adsorption affinity of the adsorbents, while the constant 1/n specifies the adsorption intensity. For the adsorption to be favorable, the value of n must be between 1 and 10 [46].

In this study, data from adsorption experiments were also fitted to thermodynamic parameters such as Gibbs free energy (∆G^0^, kJ mol^−1^), enthalpy (∆H^0^, kJ mol^−1^) and entropy (∆S^0^, kJ mol^−1^ K^−1^). These laws are generally expressed by the subsequent equation [47]

(5) $$∆G^{0}=-{\mathrm{RT}\;\ln\;k}_{C}$$

The thermodynamic parameters ∆G^0^, ∆H^0^, and ∆S^0^ can also be related by the Van’t Hoff equation, as follows [47]

(6) $$\ln k_{C}=\frac{-∆H^{0}}{R}\times \frac{1}{T}+\frac{∆S^{0}}{R}$$

where T is the absolute temperature (K) and R is the universal gas constant (8.314 J mol^−1^ K^−1^). The dimensionless parameter k_C_ is derived by multiplying the Langmuir constant (k_L_, L mg^−1^), by the molecular weight of the adsorbate (M_w_, g mol^−1^), and subsequently multiplying it by the factors 1000 and 55.5, which represent the number of moles of pure water present in one liter, as follows [48]:

(7) $$k_{C}=k_{L}\times M_{w}\times 1000\times 55.5$$

The analysis of absorption kinetics involved the utilization of various models, namely the pseudo-first-order and pseudo-second-order models, as well as intraparticle diffusion, external-film diffusion, and internal-pore diffusion models. The pseudo-first-order kinetic model can be mathematically described by the following equation [46]:

(8) $$\ln\left(q_{e}-q_{t}\right)=\ln\left(q_{e}\right)-k_{1}t$$

where k_1_ (min^−1^) is the pseudo-first-order rate constant while the terms q_e_ and q_t_ expressed in mg g^−1^ indicate the amount of MB dye adsorbed per unit weight of adsorbent at equilibrium and at any time t, respectively.

On the other hand, the pseudo-second-order kinetic model is described by the subsequent equation [46]:

(9) $$\frac{t}{q_{t}}=\frac{1}{k_{2}q_{e}^{2}}+\frac{1}{q_{e}}t$$

where k_2_ (g mg^−1^ min^−1^) is the pseudo-second-order rate constant.

Finally, the intraparticle diffusion model was used to better explain the adsorption of MB molecules on the surface of geopolymeric compounds, and to estimate the rate-limiting step of the adsorption process. This model assumes that in uniformly mixed solutions, intraparticle diffusion is generally the rate-controlling step of the process. The equation that represents this model is the following [46]:

(10) qt=k3t12+A

where the parameter k_3_ (mg g^−1^ min^−1/2^) represents the rate constant of intraparticle diffusion, while A (mg g^−1^) is a constant associated with the thickness of the boundary layer. A higher value of A indicates a more pronounced boundary layer effect. If the plot of qt against the square root of time exhibits multiple linear segments, it indicates that diffusion takes place in multiple stages throughout the process.

The internal pore diffusion model was also applied in this investigation to clarify the kinetic adsorption results. If the adsorption rate is governed by the phenomenon of particle diffusion, then it is expressed by the subsequent equation [46]:

(11) $$-\ln\left(1-{\left(\frac{q_{t}}{q_{e}}\right)}^{2}\right)=\frac{2\pi^{2}D_{p}}{r^{2}}\;t$$

However, if the adsorption rate is governed by the external-film-diffusion phenomenon, it is expressed by the subsequent equation [46]:

(12) $$-\ln\left(1-\left(\frac{q_{t}}{q_{e}}\right)\right)=\frac{D_{f}C_{s}}{{h\;r\;C}_{z}}\;t$$

where q_e_ (mg g^−1^) and q_t_ (mg g^−1^) characterize the amount of solute that the adsorbent can take up from the medium at the equilibrium and at a particular time t, respectively. The concentration of ions in the solution and adsorbent phases are represented by C_s_ (mg L^−1^) and C_z_ (mg kg^−1^), respectively, while t (time) denotes the contact time. The average radius of the adsorbent particles is indicated by r (1 × 10^−7^ m), and h represents the thickness of the film surrounding the particles, which is expected to be 10^−6^ m in slightly stirred solutions. Furthermore, D_p_ (m^2^ min^−1^) refers to the diffusion coefficient in the adsorbent phase, and D_f_ (m^2^ min^−1^) corresponds to the diffusion coefficient in the film phase surrounding the adsorbent particles.

### 2.5. Photodegradation Studies

The heterogeneous photocatalysis assays were developed following the procedure explained in our previous investigation [49]. These assays were carried out in batch systems equipped with ultraviolet light lamps at λ = 310 nm. In the experiment, the geopolymeric compounds were mixed with water at a concentration of 0.2 g L^−1^. The mixture, containing 500 mL of water at pH = 7, also contained 20 mg L^−1^ of MB dye [49]. The degradation rate of the dye for all samples was assessed using the Langmuir–Hinshelwood equation [50], which can be represented as follows [51]:

(13) $$\ln\frac{C_{0}}{C_{t}}=k\;K\;t=k_{\mathrm{app}}t$$

where k (min^−1^) and K denote the actual rate constant, and the adsorption constant of the dye on the geopolymeric compounds, respectively. The initial concentration of the dye is denoted by C_0_ (mg L^−1^), while the concentration at a precise time t (min) is denoted by C_t_ (mg L^−1^). The apparent rate constant, k_app_ (min^−1^), is estimated by plotting ln(C_0_/C_t_) against time t. The slope of the curve-fit line is the constant k_app_, and the intercept is zero.

### 2.6. Total Efficiency and Reuse

Finally, to confirm the reuse of the synthesized geopolymeric compounds for the photodegradation of MB, an experiment was carried out to evaluate their recycling potential. The reuse experiment comprised five successive treatment cycles, where at the end of each cycle, the suspensions were left to rest for at least one hour to then remove the supernatant liquid. The precipitated solids were washed with methanol containing 6% (*v/v*) acetic acid as eluent. After washing, the materials were dried and utilized in a new cycle. During each cycle, a fresh MB solution (20 mg L^−1^) was treated using 200 mg L^−1^ of geopolymeric compound. This experimental setup was based on previous research [49].

## 3. Results

### 3.1. Characterization

#### 3.1.1. XRD Analysis

**Figure 2:**
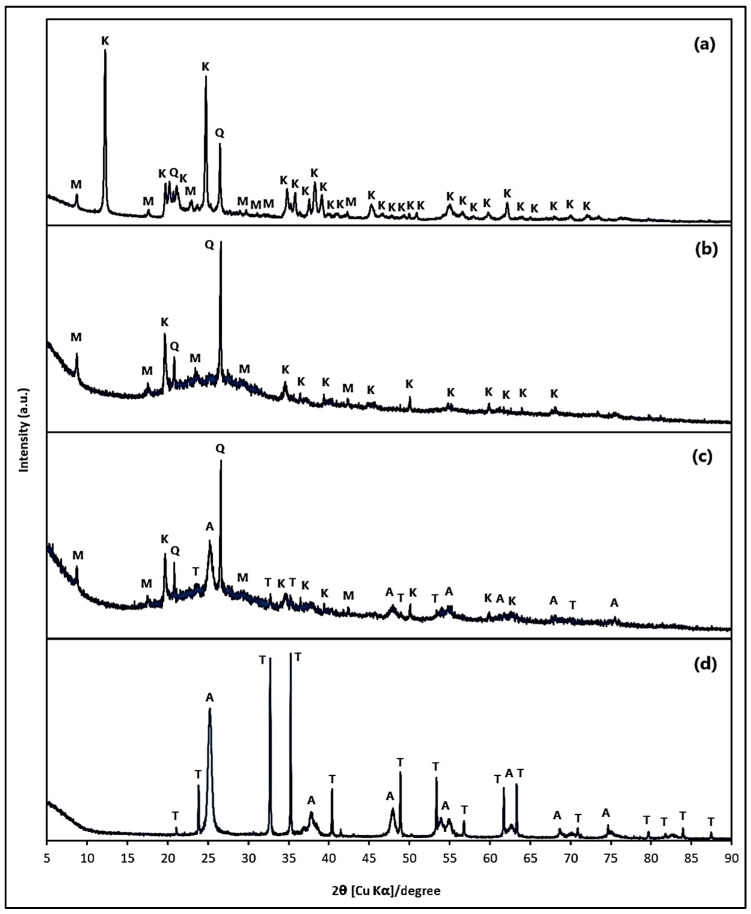
X-ray diffraction (XRD) pattern of (**a**) Kaolin, (**b**) Geopolymer, (**c**) Geopolymer/ZnTiO_3_/TiO_2_, and (**d**) ZnTiO_3_/TiO_2_. K: Kaolinite, M: Muscovite, Q: Quartz, A: TiO_2_, and T: ZnTiO_3_.

Figure 2 comparatively displays the XRD patterns of (a) Kaolin, (b) Geopolymer, (c) Geopolymer/ZnTiO_3_/TiO_2_, and (d) ZnTiO_3_/TiO_2_ semiconductor.

According to XRD analysis, kaolin is composed of 42.6% kaolinite (K), 43.6% muscovite (M), and 13.9% quartz (Q). Kaolinite was identified as an orthic phase with unit cell parameters of a = b = 5.16 Å and c = 7.41 Å, belonging to space group P1(1) according to the standard COD card No. 96-900-9231. On the other hand, Muscovite was determined to be monoclinic phase with unit cell parameters of a = 5.18 Å, b = 8.99 Å, and c = 20.23 Å, and space group C12/c1(15) according to the standard COD card No. 96-900-1058. Finally, quartz was found to have a hexagonal phase with unit cell parameters of a = b = 4.94 Å, and c = 5.42 Å, and space group P3121(152) according to the standard COD card No. 96-900-5021. In Figure 2d, the main diffraction peaks of the ZnTiO_3_ and TiO_2_ semiconductors are displayed. According to XRD analysis, the heterogenous semiconductor consists of 53% ZnTiO_3_ (T), and 47% TiO_2_ (A). ZnTiO_3_ was determined to have a rhombohedral phase with unit cell parameters of a = b = 5.08 Å and c = 13.93 Å, belonging to space group R-3(148) according to the standard COD card No. 00-026-1500, while TiO_2_ (anatase phase) was indexed as a tetragonal phase with unit cell parameters of a = b = 3.79 Å and c = 9.51 Å, and space group I41/amd(141) according to the standard COD card No. 96-900-9087. The crystal size of the ZnTiO_3_/TiO_2_ particles (27.61 ± 3.79 nm) was determined by measuring the main peak of the respective diffraction patterns using the well-known Scherrer equation [52].

Furthermore, from the XRF analysis, the main oxides present in the kaolin and the geopolymeric compounds are listed in Table 1.

#### 3.1.2. SEM-EDX Analysis

**Figure 3:**
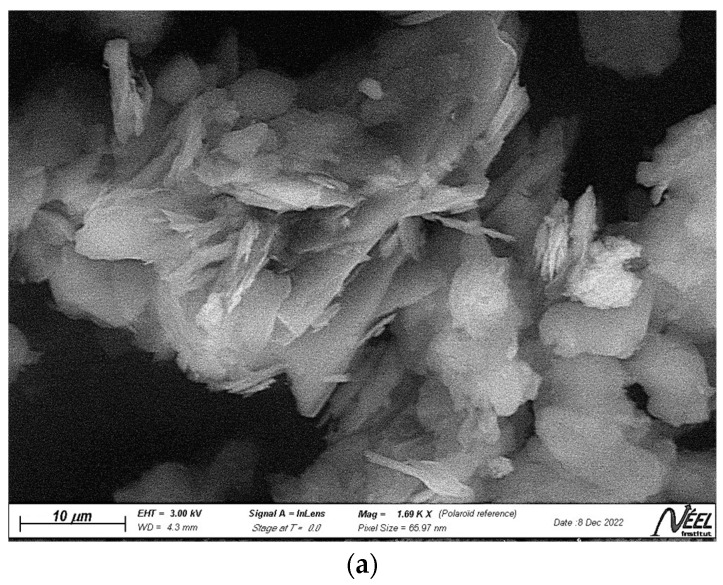

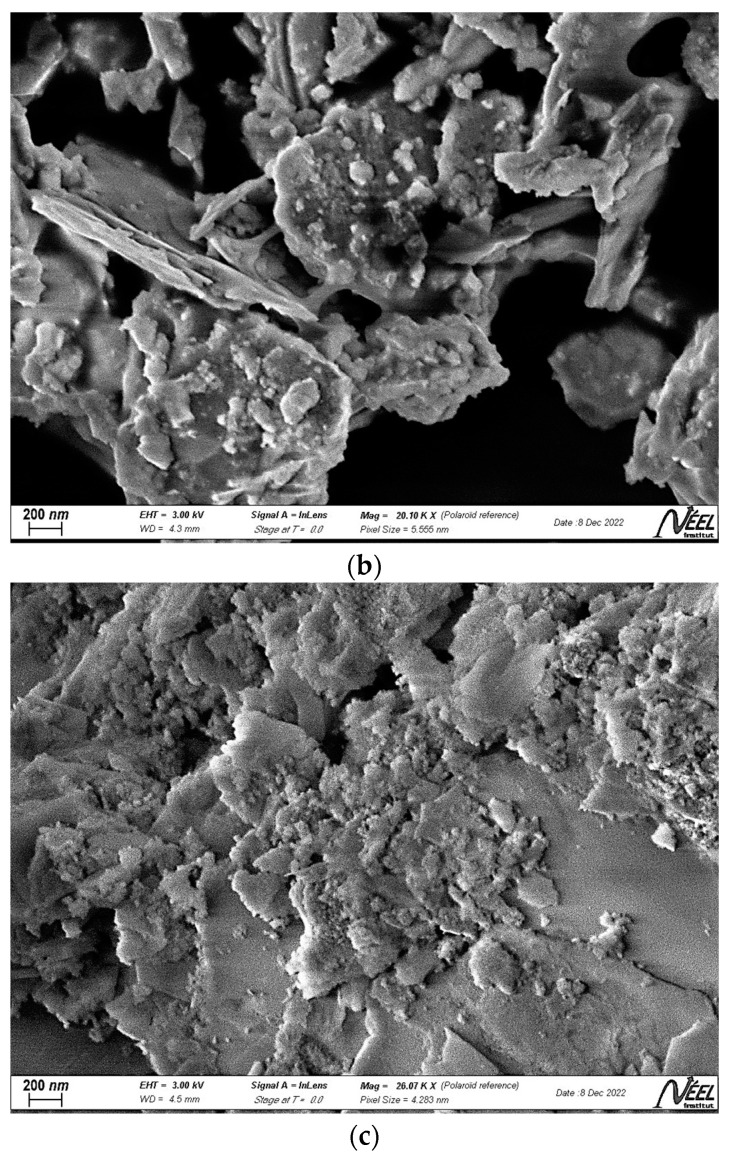
Scanning Electron Microscopy (SEM) images of (**a**) Kaolin, (**b**) GP, and (**c**) GTA.

Figure 3a–c displays the photomicrographs of kaolin, geopolymer (GP) and geopolymer/ZnTiO_3_/TiO_2_ (GTA), respectively. The kaolin surface appears as a three-dimensional structure composed of small particles or flat sheets, while the geopolymer surface has a porous and rough texture, which gives it a high specific surface area and adsorption capacity for chemical substances. On the other hand, the geopolymer impregnated with the ZnTiO_3_/TiO_2_ hybrid semiconductor has a very irregular appearance with spherical particles of the semiconductor agglomerated on a rough surface. The average particle size estimated from Figure 3c for the ZnTiO_3_/TiO_2_ particles was 31.46 ± 4.15 nm.

**Figure 4:**
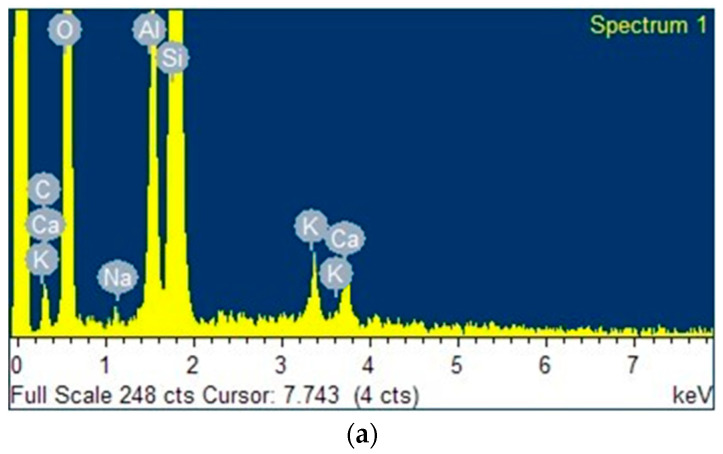

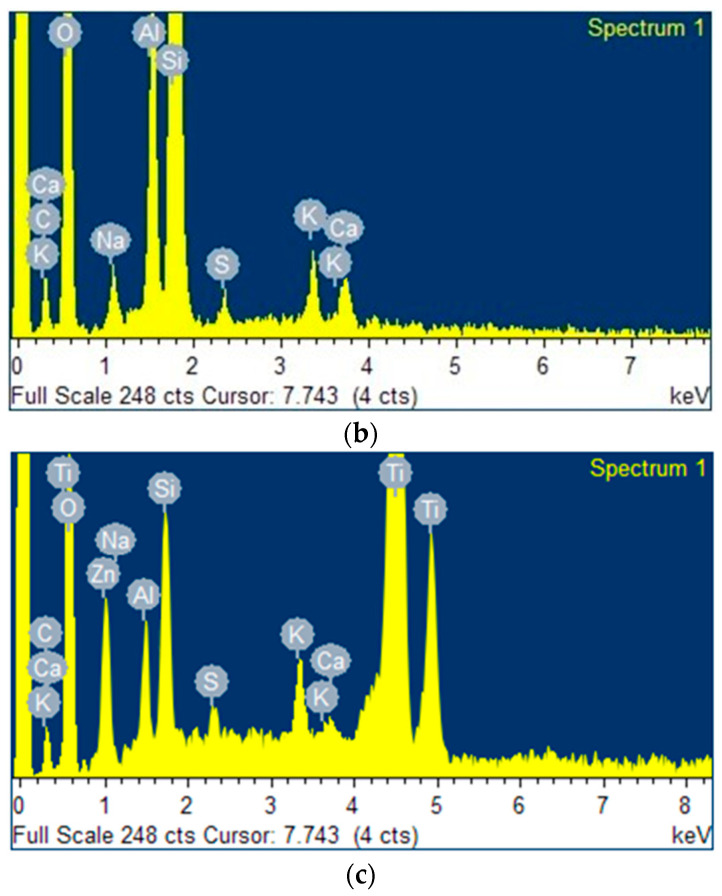
Energy Dispersive X-ray (EDX) spectra of (**a**) Kaolin, (**b**) GP, and (**c**) GTA.

Figure 4a–c presents the spectra of kaolin, geopolymer (GP) and geopolymer/ZnTiO_3_/TiO_2_ (GTA), respectively. All samples have the exchange cations Ca and K. In addition, GP and GTA have the elements Na and S, which were incorporated as salts during the polymerization process. Finally, GTA has a high amount of Zn and Ti, due to the presence of the ZnTiO_3_/TiO_2_ semiconductor.

Table 2 shows the percentage composition (wt.%) of the elements present in the kaolin, GP and GTA samples. These values corroborate the results shown in the EDX spectra of Figure 4.

#### 3.1.3. Point of Zero Charge (PZC) and Specific Surface Area (SSA) Analysis

The point of zero charge (pH_PZC_) and the SSA (m^2^ g^−1^) of the samples in powder form as well as pellets are listed in Table 3. As this table shows, kaolin has relatively low area values. The geopolymerization process allowed the SSA of the kaolin to increase, which is consistent with the results achieved by scanning electron microscopy (SEM). In addition, samples in the form of pellets show a lower specific surface area related to samples in powder form due to the procedure utilized for their preparation. On the other hand, Table 3 also displays that the pH_PZC_ of the geopolymer-based samples is on average 7.0.

Although powdered samples exhibit higher specific surface areas, pelletized samples have higher mechanical and chemical resistance. Therefore, in this investigation, geopolymeric compounds in the form of pellets were preferred for the adsorption and photodegradation of the MB dye. In this way, easy handling and recovery of the pelletized geopolymer samples was accomplished at the conclusion of the removal procedure, enabling their subsequent reuse after several treatment cycles.

### 3.2. Adsorption Studies

#### 3.2.1. Effect of Solution pH

**Figure 5:**
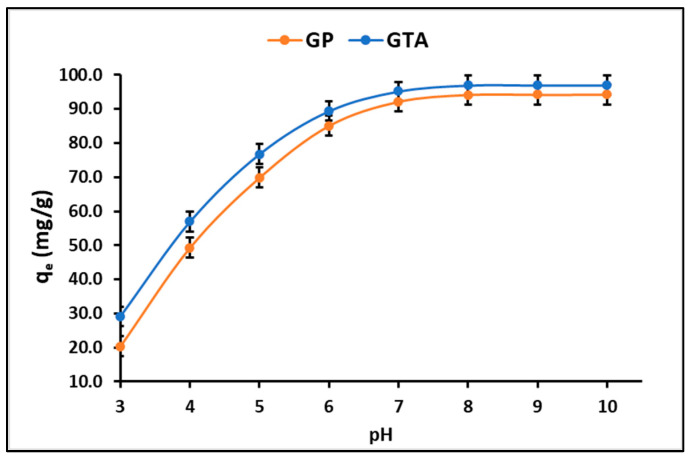
Effect of pH on the adsorption of dye on GP and GTA.

The results of the dye adsorption experiment as a function of the pH of the solution are presented in Figure 5. The geopolymeric compounds exhibited pH_PZC_ values approximately at 7.0. As a result, when the pH surpasses the pH_PZC_, the surface carries a net negative charge, facilitating the adsorption of the cationic dye molecule. Conversely, at pH levels below the pH_PZC_, the adsorption of the dye diminishes due to the net positive charge on the surface, leading to electrostatic repulsion.

After observing a minimal rise in dye adsorption in the solution when pH values exceeded 8.0, it was decided that the optimal operational conditions for the adsorption and photodegradation experiments was at pH 7.0.

#### 3.2.2. Effect of Initial Dye Concentration

**Figure 6:**
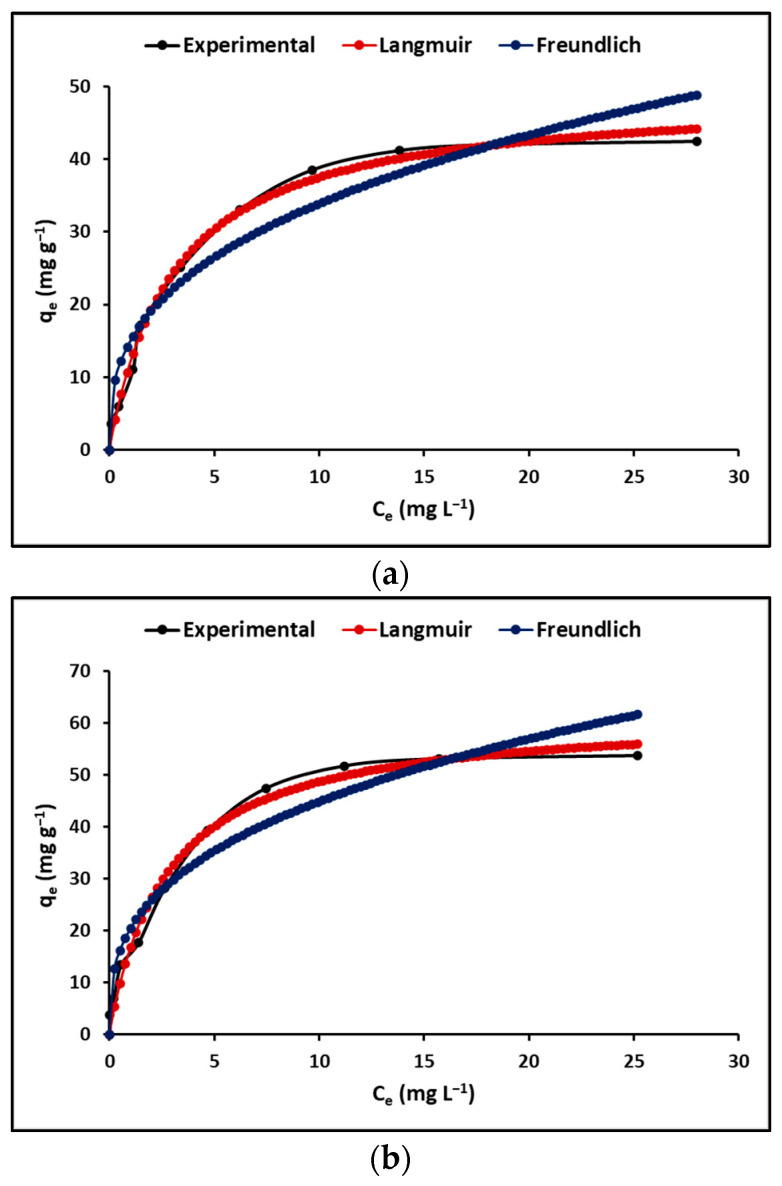
Absorption isotherms of (**a**) GP, and (**b**) GTA.

This study analyzed the Langmuir and Freundlich isotherms as equilibrium models that depend on the initial concentration of the adsorbate. The Langmuir isotherm assumes a uniform and homogeneous surface, where homogeneous and precise sites are accessible for adsorption, generating limited interaction between molecules. On the other hand, the Freundlich equation does not suppose uniformity in the energy of the surface sites, which reflects a heterogeneous surface with unlimited adsorption capacity. Figure 6 exhibits the adsorption isotherms of the MB molecule for the GP and GTA compounds. From this figure, it is clear that the Langmuir model is better than the Freundlich model in describing the behavior of both samples.

Table 4 provides the determined values of the Langmuir and Freundlich constants at three temperatures (293.15, 298.05, and 303.15 K). The table exposes that the values of the R_L_ equilibrium parameter or separation factor oscillates from zero to one, whereas the values of the coefficient n, which indicates the adsorption intensity, oscillates from one to ten. These results suggest that the adsorption of MB dye on the surface of the samples was satisfactory.

#### 3.2.3. Effect of Temperature

**Figure 7:**
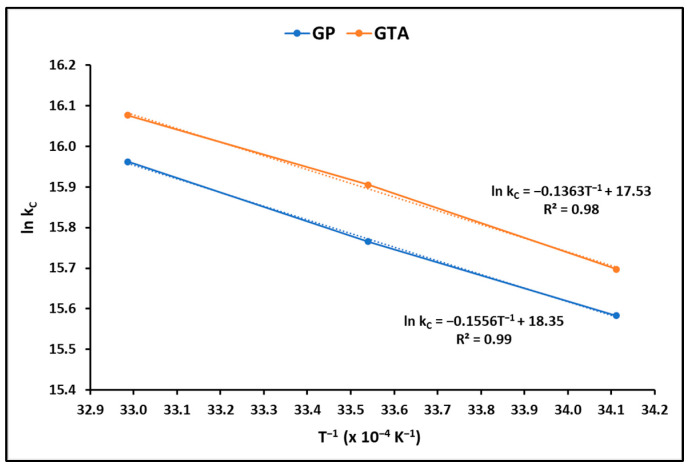
Thermodynamic study of MB adsorption on GP and GTA pellets.

The thermodynamic parameters, including the Gibbs free energy change (∆G°), enthalpy change (∆H°), and surface entropy change (∆S°), provide valuable insights into the spontaneity and feasibility of a process. To calculate these parameters, the equilibrium constant was assessed at various temperatures, as illustrated in Figure 7.

The results of the thermodynamic parameters calculated in this investigation are displayed in Table 5. On average the Gibbs free energy (∆G°), enthalpy (ΔH°) and entropy (ΔS°) were estimated at −39.25 kJ mol^−1^**,** −27.96 kJ mol^−1^ and 0.22 kJ mol^−1^ K^−1^, respectively, which supports the spontaneous and exothermic behavior of the MB uptake by geopolymeric compounds.

#### 3.2.4. Effect of Contact Time

**Figure 8:**
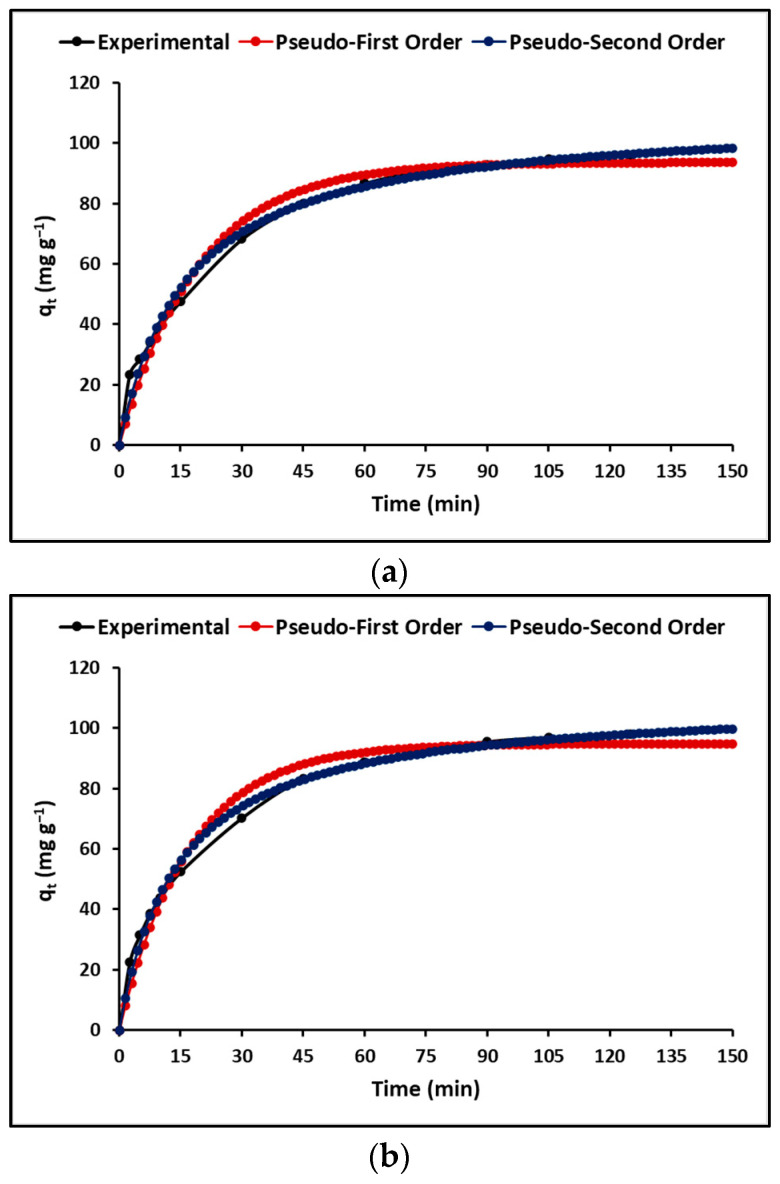
Adsorption kinetics of (**a**) GP, and (**b**) GTA.

In this study the Lagergren (pseudo-first order) and Ho (pseudo-second order) models were applied to explain the effect of contact time or adsorption kinetics. As shown in Figure 8, both models reveal a fast initial adsorption phase followed by an equilibrium stage. As indicated in Table 6, the pseudo-second order model displays a higher correlation coefficient than the pseudo-first order model, indicating a chemisorption process [53].

**Figure 9:**
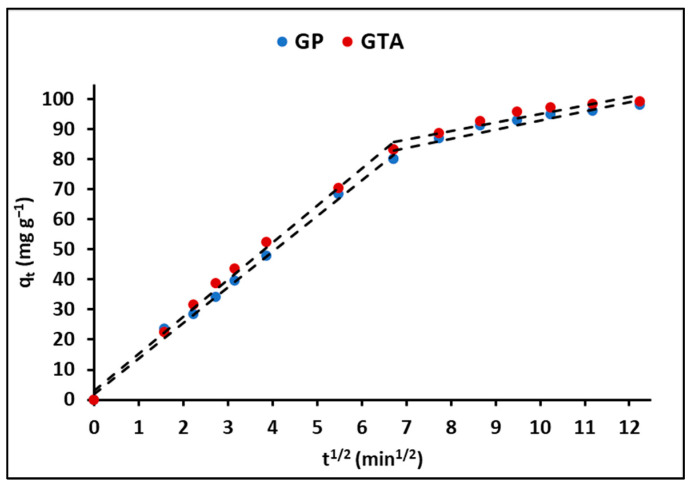
Intra-particle diffusion plots for MB adsorption by GP and GTA.

The intraparticle diffusion model was employed to elucidate the adsorption rate, taking into account the transfer rate of MB molecules from the aqueous solution to the adsorption sites on the geopolymeric compounds. Figure 9 exhibits the evolution of the q_t_ (mg g^−1^) curves in relation to the square root of time (t^1/2^) for both the GP and GTA compounds. The figure demonstrates that the adsorption process can be divided into two distinct linear regions. Hence, the MB adsorption process can be described as a combination of film diffusion initially, followed by a subsequent particle diffusion process [54].

Table 6 displays the MB adsorption kinetic parameters calculated in this study for the geopolymeric compounds.

### 3.3. Photocatalytic Studies

**Figure 10:**
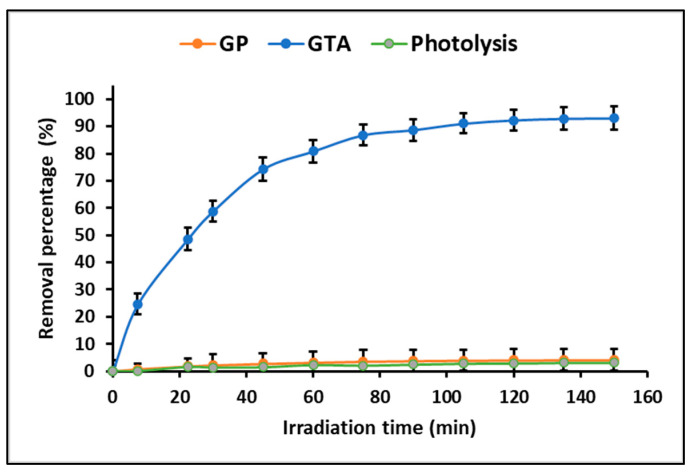
Photocatalytic dye degradation by GP and GTA.

ZnTiO_3_/TiO_2_ is a hybrid photocatalyst that is extensively employed for efficient degradation of organic compounds due to its high photooxidant capacity. In this study, the kinetics of MB photodegradation under ultraviolet radiation was assessed using the Langmuir-Hinshelwood equation. This equation demonstrated a linear correlation between ln(C_0_/C_t_) and t, supporting the idea that the photodegradation of the MB dye follows a pseudo-first order reaction. The apparent rate constants (k_app_) were determined to be 0.003, and 0.018 min^−1^ for GP and GTA, respectively. These results are in agreement with those reported by other authors [51,55]. Figure 10 depicts that both samples achieved the maximum percentage of MB degradation within approximately the initial 90 min, after which the photodegradation appeared to plateau. While existing literature suggests that there is a degradation limit for contaminants through photocatalysis [56], this study achieved near-complete photodegradation of the MB dye using GTA (93%). Conversely, GP exhibited negligible photocatalytic activity (4.0%), likely attributed to the photolysis effect.

### 3.4. Total Efficiency and Reuse

**Figure 11:**
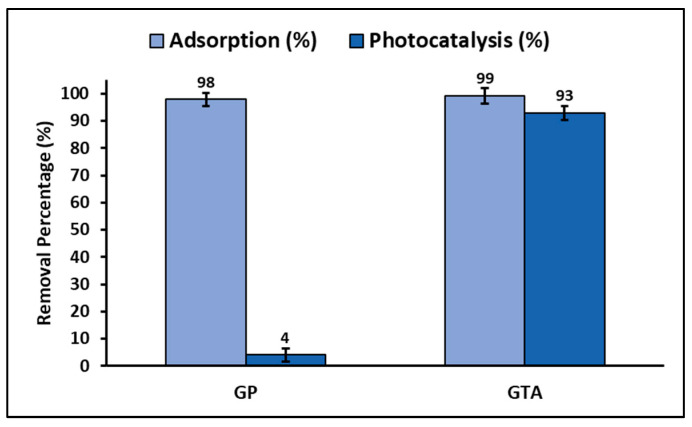
Percentage of dye adsorbed and photodegraded by GP and GTA.

The amount (%) of MB dye adsorbed and photodegraded by GP and GTA is presented in Figure 11. It is evident from this comparative figure that the GTA sample has a higher capacity for adsorption and photodegradation of the MB dye than the GP sample.

**Figure 12:**
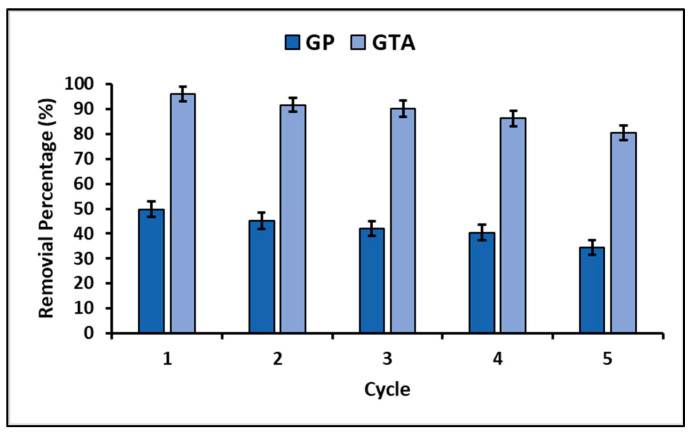
Reuse of GP and GTA.

Finally, the stability and reusability of adsorption and photocatalytic materials are critical factors to consider for their large-scale implementation. Therefore, in this investigation an experiment was performed to assess the reuse potential of geopolymeric compounds during five successive MB dye removal cycles. The results obtained are represented in Figure 12.

## 4. Discussion

### 4.1. Characterization

#### 4.1.1. XRD and XRF Analysis

**Figure 13:**
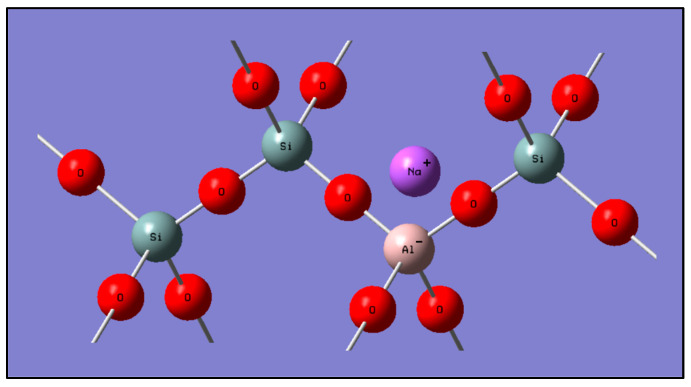
Na-poly(sialate–disiloxo) structure.

There are numerous starting materials rich in silica and alumina available for utilization as precursors in the process of geopolymerization [57]. One of these materials is kaolin, which is a predominant material in the earth’s crust. In this study, GP and GTA geopolymeric compounds were prepared from kaolin, which was first calcined to obtain metakaolin (MK). The diffraction patterns of the kaolin starting material, GP and GTA compounds, as well as the ZnTiO_3_/TiO_2_ hybrid semiconductor that was incorporated only into the GTA compound are shown in Figure 2. As shown in Figure 2a, the kaolin mineral mainly consisted of the kaolinite (K), muscovite (M) and quartz (Q) phases. Figure 2b,c show the decrease in the intensity of various peaks, which is due, on the one hand, to the formation of metakaolin by the dehydroxylation of kaolin as a result of the thermal treatment carried out, and on the other hand, to the alkaline treatment for the dissolution of the crystalline phases and subsequent geopolymerization reaction. Furthermore, in Figure 2c the incorporation of the hybrid semiconductor into the GTA geopolymeric compound is evident. Regarding the XRF analysis, Table 1 shows the composition (wt.%) of the main oxides present in the kaolin sample, and in the polymeric compounds. This table indicates that kaolin is basically formed by SiO_2_ and Al_2_O_3_ with a Si/Al ratio of 2.89. After the alkaline activation process, this relationship showed a change, being 3.05 for the geopolymeric compounds. Evidence from the literature shows that based on Si/Al ratios, geopolymeric materials can be composed of network structures of (Na, K)-poly(sialate) (–O–Si–O–Al–O–)*n*, (Na, K)-poly(sialat-siloxo) (–O–Si–O–Al–O–Si–O)*n*, and (Na, K)-poly(sialate-disiloxo) (O–Si–O–Al–O–Si–O–Si–O–)*n* [39], consequently in this study, with an Si/Al ratio of approximately 3.05, it was suggested that the geopolymeric compounds could assume the structure (Na, K)-poly(sialate-disiloxo) (O–Si–O–Si–O–Al–O–Si–O–)*n*, which is shown in Figure 13 [35,58].

#### 4.1.2. SEM-EDX Analysis

Scanning electron microscope (SEM) images of kaolin and geopolymeric compounds are shown in Figure 3. The images show that the morphology of the surface of geopolymeric compounds changes with respect to the original morphology of the kaolin surface. In fact, the surface appears more irregular and rougher in the GP and GTA composites than in pristine kaolin. The alteration in morphology, which undoubtedly contributes to the augmentation of the specific surface area, stems from both the heat treatment employed to obtain metakaolin and the subsequent process of geopolymerization. The elemental composition of kaolin and of GP and GTA geopolymeric compounds was characterized through EDX analysis. According to Figure 4, the majority elements in these samples are Al, Si and O. In addition, in the GTA compound, Ti and Zn are also observed as majority elements due to the presence of the hybrid photocatalyst. In Table 2 an increase in the Na content can be seen, as well as the occurrence of S in the geopolymeric compounds with respect to pristine kaolin, which is due to the incorporation of sodium hydroxide (NaOH) and sodium dodecyl sulfate (NaC_12_H_25_SO_4_) during the geopolymerization process. Finally, K and Ca were identified as minority elements in the three samples.

#### 4.1.3. Specific Surface Area (SSA) and Point of Zero Charge (PZC) Analysis

The geopolymeric compounds in powder form have a relatively higher SSA compared to kaolin (Table 3), this is probably due to the alkaline treatment during the geopolymerization process, which allowed the dissolution of aluminates and silicates from the mineralogical phases that constitute the pristine kaolin. On the other hand, the table clearly demonstrates a decrease in the specific surface area of the geopolymeric compounds when in pellet form compared to their powdered counterparts, primarily attributable to the extrusion process. Despite the potential limitation of reduced availability of active sites associated with a lower SSA, the pelletized compounds were utilized in this study for the adsorption of the MB dye from aqueous solutions. This selection was based on the mechanical and chemical resistance of the structured materials, which allows their easy recovery at the end of a process as well as their respective reuse. Table 3 also shows that due to the alkaline treatment, there was an increase in the pH_PZC_ of the geopolymeric compounds (pH_PZC_ ≈ 7.0) with respect to pristine kaolin (pH_PZC_ = 4.6).

### 4.2. Adsorption Studies

#### 4.2.1. Effect of Solution pH

The findings of this study provide compelling evidence that the adsorption capacity of geopolymeric materials for MB is strongly influenced by the pH of the solution. This dependence arises from its impact on both the speciation of the dye in the solution and the distribution of surface charges on the materials [59]. Consequently, the pH plays a crucial role in modulating the electrostatic interactions, whether attractive or repulsive, between the MB species present in the solution and the surface of the synthesized geopolymeric materials. Based on the literature, the MB molecule can exist in an aqueous solution in two forms: as undissociated molecules (MB°) and as cationic species (MB^+^). Therefore, at pH = 3, the MB° form of MB is the predominant species (86%), at pH = pKa = 3.8, both the MB° and MB^+^ coexist in equal proportions (50%), and at pH > 6, the cationic form (MB^+^) is practically the only species present [60].

The MB adsorption capacity of geopolymeric compounds increases dramatically when the pH increases from 3.0 to 7.0 (Figure 5). However, at pH values > 7.0 the adsorption capacity tends to be constant. The elevated adsorption capacity observed at alkaline pH values can be attributed to the rise in hydroxyl ions, which results in increased electrostatic attraction between the predominant cationic species of MB^+^ and the negatively charged surface of geopolymeric compounds [61]. Nevertheless, at extremely high alkaline pH levels, the OH^−^ ions may form complexes with other ions, potentially influencing the adsorption of the dye on the surface of the adsorbent [62]. This phenomenon could result in the precipitation of dye molecules on the adsorbent surface. Consequently, the adsorption mechanism at alkaline pH is likely to be a combination of electrostatic attraction and precipitation [63]. On the other hand, Figure 5 also shows that the geopolymeric compounds presented a reasonably good MB^+^ adsorption capacity at pH < 7, where electrostatic interactions do not favor adsorption. Under these experimental conditions (pH < 7), it is suggested that the adsorption of the dye could occur by ionic exchange, since this cationic MB^+^ species would be competing with H^+^ for the active sites of the geopolymeric compounds [64].

#### 4.2.2. Effect of Initial Dye Concentration

As seen in Figure 6, the GP and GTA geopolymeric compounds initially show a rapid increase in the MB removal rate from the solution, after which the MB removal rate stabilizes. With an increase in dye concentration, a greater number of dye molecules compete for the limited available active sites on the surface of geopolymeric compounds. The limited availability of active sites on the surface of geopolymeric compounds causes them to rapidly become saturated as the concentration of MB increases in the solution. Consequently, the findings from this study indicate that the initial concentration of MB dye plays a crucial role in overcoming the resistance to mass transfer of dye molecules from the aqueous solution to the surface of the geopolymeric compounds. [65]. Table 4 shows that for both GP and GTA, the Langmuir isotherm model offers a better fit to the experimental data than the Freundlich isotherm model. Therefore, it is suggested that the adsorption of MB molecules on the homogeneous surface of these compounds occurred as a monolayer adsorption rather than a multilayer adsorption [66]. Additionally, during the adsorption process, the dye molecules have the potential to move through the channels and pores of the geopolymeric compounds, thereby displacing the exchangeable cations that are initially present within these materials. These results are similar to those reported by other authors [39]. Finally, Table 4 shows the values of the Langmuir (R_L_) and Freundlich (n) constants that confirm the favorable adsorption of the MB dye onto the geopolymeric compounds synthesized in this study.

#### 4.2.3. Effect of Temperature

The adsorption of MB dye is influenced by temperature, as it determines the thermodynamic equilibrium between the dye adsorbed on the surface of GP and GTA and the dye present in the aqueous solution. [67]. To analyze the effect of temperature on the MB adsorption capacity of GP and GTA geopolymeric compounds, adsorption isotherms were determined at temperatures of 293.15 K (20 °C), 298.15 K (25 °C), and 303.15 K (30 °C) and pH = 7 ± 0.2 (Table 4). On the other hand, Table 5 displays the thermodynamic parameters obtained in this study for the removal of the MB dye. The negative values of ∆G° suggest the spontaneity and feasibility of MB adsorption on the GP and GTA geopolymeric compounds. The decrease in the magnitude of ∆G° with increasing temperature indicates an enhancement in the efficiency of the adsorption process at higher temperatures. Moreover, Table 5 shows positive values of ∆H° and ∆S°, indicating that the adsorption of the MB dye occurred endothermically. The positive ∆S° suggests an increase in randomness at the solid/solution interface, with some modifications in the structure of the active sites of the geopolymeric compounds during dye adsorption.

#### 4.2.4. Effect of Contact Time

Although isothermal models of adsorption allow inference of the efficiency of the process, it is also important to determine the adsorption kinetics. The kinetic models of adsorption in Figure 8 represent the necessary time of adsorbent/adsorbate contact for the complete adsorption of the MB dye onto the geopolymeric compounds GP and GTA. This figure shows that the MB concentration decreases rapidly at the beginning of the adsorption process, but after 60 min it tends to be constant for both geopolymeric compounds. The rapid initial stage of adsorption is due to both the high availability of vacant sites for adsorption and the presence of a high concentration gradient. Table 6 presents the kinetic constants and correlation coefficients calculated for the pseudo-first-order and pseudo-second-order equations. The results demonstrate that the adsorption of MB onto GP and GTA compounds is most accurately described by the pseudo-second-order kinetic model, indicating a chemical interaction process. In this process of chemical interaction, the MB molecule could share and exchange electrons with the functional groups of the geopolymeric compounds prepared from metakaolin (MK). These results are similar to those reported by other authors [68,69]. According to the literature, the poly(sialate-disiloxo) geopolymer network consists of (SiO_4_) and (AlO_4_) groups connected by covalent Si–O–Al– bond. The presence of cations such as Na^+^, K^+^, Ca^2+^ within the structural cavities of the poly(sialate-disiloxo) balance the negative charge of Al^3+^ in coordination (IV) [39]. During the adsorption process, the removal of the MB dye by geopolymeric compounds, can be explained by ionic exchange and the consequent interactions between the positive charge of (MB^+^) and the negative charge of tetrahedral Al (–Si–O–Al–O–Si–O) in geopolymeric compounds [39]. This mechanism would explain the chemisorption of the MB dye by geopolymeric compounds GP and GTA.

Furthermore, Figure 9 reveals the presence of two linear regions when fitting the experimental data to the intraparticle diffusion model. This finding suggests that the adsorption process of MB can be described by a combination of external film diffusion, followed by internal pore diffusion. Table 6, in addition, provides a comprehensive summary of the linear regression analysis conducted for the diffusion kinetic models. The diffusion of the external film displayed the highest regression coefficient (R^2^) values among the models evaluated. Moreover, the relatively high values of A suggest that surface adsorption is the limiting step in the overall adsorption process [70].

### 4.3. Photocatalytic Studies

The evidence from this study demonstrates that the photocatalytic mechanism of geopolymeric compounds is extrinsic [71]; that is, it depends on the presence of the ZnTiO_3_/TiO_2_ hybrid semiconductor. Therefore, Figure 10 shows that the GTA compound presents a high photocatalytic activity (93%), while in the GP compound the photocatalytic activity (4%) is probably due to the phenomenon of photolysis. The action of light on the ZnTiO_3_/TiO_2_ semiconductor immobilized in the geopolymer allows an electron to be promoted from the valence band (VB) to the conduction band (CB), generating an electron/hole pair (e^−^/h^+^). Photoexcitation takes place solely when the energy of the incident photon is equal to or greater than the bandgap of the semiconductor [72]. Previous studies indicate that the ZnTiO_3_/TiO_2_ hybrid semiconductor has a bandgap energy of 3.07 eV, lower than that of TiO_2_ (3.12 eV), so its photoactivity moves to the visible range [73]. In the presence of an aqueous medium, the photogenerated e^−^/h^+^ pair can react to produce HO˙ radicals, which are extremely oxidizing agents and can easily degrade organic pollutants such as the MB dye [74]. At the same time, the holes (h^+^) in the VB can react with oxygen to form anionic superoxide radicals, ˙O_2_^−^. These species, in addition to being oxidizing agents, can also prevent recombination of pairs (e^−^/h^+^). The superoxide radical when protonated forms the hyperoxyl radical, HO_2_˙, and if both radicals combine, they can form H_2_O_2_ + O_2_, or two highly reactive hydroxyl radicals HO^−^ + O_2_. All these reactive oxygen species (ROS) are highly reactive and can oxidize various organic compounds, including the MB dye, degrading them to H_2_O and CO_2_ [75,76].

### 4.4. Total Efficiency and Reuse

This study found that the geopolymeric compounds GP and GTA were highly efficient in the adsorption of MB dye from aqueous solutions. However, the addition of the ZnTiO_3_/TiO_2_ hybrid semiconductor to the geopolymer to obtain the GTA compound, allowed improvement of the efficiency of the removal process through photodegradation of the adsorbed dye. Thanks to the combination of adsorption and photocatalysis processes, the dye molecules that initially adsorbed and accumulated on the surface of the GTA compound were the first to degrade under irradiated light when the photocatalytic process started. Consequently, the continuous migration and subsequent photo-oxidation occurring on the surface of the GTA compound significantly contributed to enhancing the removal efficiency at the solid–liquid interface. This process generated a concentration gradient that served as the primary driving force for the removal of MB from the aqueous solution. In summary, the GTA compound exhibited a more effective performance than the GP compound for the removal of MB dye from aqueous solutions by coupling “adsorption–photodegradation” processes [77].

On the other hand, in this study, the efficacy of the reuse of geopolymeric compounds was evaluated through five consecutive treatment cycles, with the purpose of estimating their chemical and mechanical stability. Figure 12 shows the results of the experiment, which indicate that the percentage of removal of the MB dye decreases with each cycle. Despite this, after five cycles, the loss of dye removal capacity for both geopolymeric compounds did not exceed 15%. The observed decreased efficacy may be due to the chemical adsorption of dye molecules onto the surface of compounds, reducing the availability of active sites. However, XRF analysis confirmed that there were no significant changes in the original composition of the compounds after the fifth cycle. Therefore, GP and GTA geopolymeric compounds are chemically stable and maintain adequate activity during five treatment cycles, effectively removing MB dye by the combination of two processes, adsorption and photocatalysis. Regarding the mechanical stability of the compounds tested, in this study, it was verified that the materials adapted to the form of pellets were mechanically stable and demonstrated effectiveness in removing the MB dye, which suggests its effective application in wastewater remediation processes.

The findings of this study demonstrate that geopolymeric materials serve as valuable supports for photocatalysts, as they enable the adsorption of dyes for subsequent photodegradation. Table 7 provides a summary of the MB adsorption capacity of the geopolymeric composites synthesized in this study, along with a comparison with other materials reported in the literature.

Compared to the listed materials, geopolymer (GP) and geopolymer/ZnTiO_3_/TiO_2_ (GTA) composites have intermediate adsorption capacity for MB dye. In particular, the GTA presents a higher adsorption capacity than the GP, which suggests that the incorporation of ZnTiO_3_/TiO_2_ in the geopolymer can improve its adsorption capacity. However, compared to some highly effective adsorption materials such as chitosan clay microspheres, geocomposites still have a way to go to reach equally high adsorption capacities. Compared to other geopolymeric materials, both GP and GTA have higher adsorption capacities, being surpassed only by the materials hierarchical xNGeo particle (115) and y Py-GP4 porous geopolymer-based pyrophyllite. Even so, these results indicate that geopolymeric composites are a promising option as adsorption materials for the removal of pollutants from wastewater. It is important to note that these results are specific to the adsorption of MB dye, and that the adsorption capacity of the materials may vary for other contaminants. Additionally, factors such as particle size, pore structure, and chemical composition of materials can affect their adsorption capacity and must be considered when selecting an adsorption material for a specific contaminant.

Likewise, the geopolymeric compound GTA, synthesized in this study, has shown effective capability in the photodegradation of dyes in aqueous effluents. Table 8 provides a summary of the operational and process conditions employed in various research studies that conducted the photodegradation of MB using different semiconductors based on titanium oxide. The conditions are characterized by four factors: the initial concentration of the MB dye, the type of light used for irradiation, the reaction time, and the efficiency of MB removal.

The photocatalytic degradation efficiency of MB is an important parameter to evaluate the capacity of photocatalytic materials for the removal of contaminants in water. The comparison of the results shows that the GTA has a significantly higher degradation efficiency than the GP. While GTA has a photocatalytic degradation efficiency of 93%, the degradation efficiency of GP is very low (4%) for MB. The difference in photocatalytic degradation efficiency between GTA and GP can be attributed to the presence of ZnTiO_3_/TiO_2_ hybrid semiconductor in the GTA compound, which acts as catalysts and improves photodegradation efficiency. On the other hand, GP shows a very low degradation efficiency for methylene blue, indicating that it has a very limited photodegradation capacity due to the lack of the photocatalyst. The GTA geopolymeric compound has a photodegradation efficiency comparable to other materials such as TiO_2_-coated geopolymer spheres and TBBFS/geopolymer which also have a percentage of 93%. On the other hand, fly ash-based geopolymer and geopolymer/zeolite present slightly lower percentages, while the geopolymeric compounds geopolymer/zeolite doped with TiO_2_, perlite-based geopolymer, and RMGP have higher efficiency for the photodegradation of MB than the GTA compound. Even so, the results of this study indicate that the GTA compound is a promising alternative as a photocatalytic material for the removal of pollutants from wastewater.

### 4.5. Proposed Mechanism of Removal of the MB Dye

The removal of the methylene blue dye from aqueous solutions by the porous geopolymeric compounds GP and GTA can be explained based on the adsorption and photodegradation mechanisms. Both the GP and the GTA compounds present adsorption properties due to their porous structure and the presence of active sites on their surface. Methylene blue, as a positively charged organic compound, can adsorb to the surface of these materials through electrostatic and van der Waals interactions. The functional groups present in both materials can interact with the functional groups of methylene blue, which facilitates its adsorption. In addition to adsorption, the GTA compound is also capable of degrading methylene blue through photocatalytic processes. The GTA compound contains ZnTiO_3_ and TiO_2_ particles, which act as photocatalysts. When the material is illuminated with ultraviolet light, the valence electrons of the photocatalysts (ZnTiO_3_ and TiO_2_) are excited to higher energy levels. These excited electrons can be transferred to the adsorbed methylene blue, generating reactive oxygen species (ROS), such as hydroxyl (HO˙) and superoxide (−O_2_^−^) radicals. These ROS are highly reactive and can attack and degrade methylene blue, breaking its bonds and oxidizing functional groups, leading to its degradation and mineralization.

**Figure 14:**
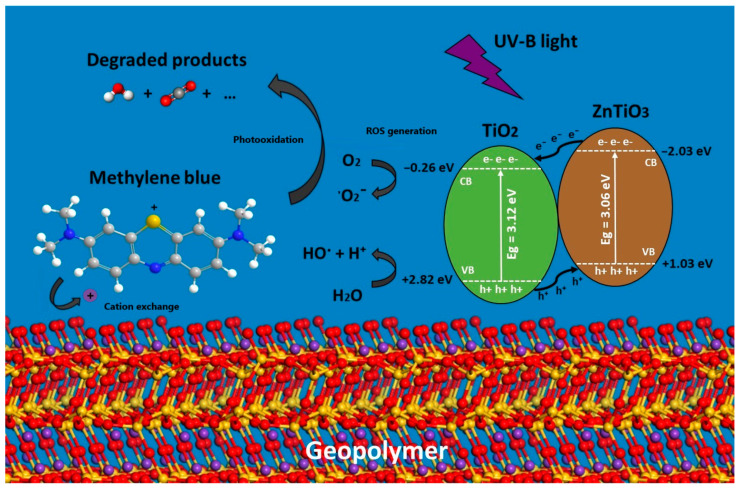
Proposed mechanism of removal of the MB dye by the GTA compound.

As can be seen in Figure 14, the combination of adsorption and photodegradation on the GTA compound creates a synergy that improves the methylene blue removal efficiency. The initial adsorption of methylene blue on the surface of this compound allows greater contact between the contaminant and the photocatalysts, thus improving the efficiency of photodegradation. Furthermore, adsorption can help prevent the release of adsorbed contaminants back into the environment during the photodegradation process. Therefore, the combination of adsorption and photodegradation is a good strategy since it allows a higher efficiency in the removal of methylene blue, thus contributing to its potential application in the treatment of wastewater contaminated with this compound.

In summary, the results clearly show that GTA has much higher photocatalytic degradation efficiency than GP. The presence of ZnTiO_3_/TiO_2_ in the GTA increases the adsorption capacity and the active sites available for MB photodegradation, thus improving the efficiency of photodegradation.

## 5. Conclusions

This research presents the use of geopolymeric compounds for the adsorption and photodegradation of the methylene blue (MB) dye in aqueous solutions. The results obtained indicate that these porous compounds are highly efficient in removing the dye. In addition, the incorporation of the ZnTiO_3_/TiO_2_ hybrid photocatalyst in the geopolymeric matrix allowed the GTA compound to be obtained, which showed high efficiency in the photodegradation of the MB dye in the presence of UVB light. Likewise, the combination of adsorption and photocatalysis was shown to be an effective strategy for the removal of the MB dye. In fact, the research demonstrated that the removal of the MB dye is controlled by a combination of ion exchange, precipitation, and photooxidation.

The novelty of this study lies in the synthesis and characterization of the porous geopolymeric compounds, in particular the GTA compound, which has not been previously investigated in the scientific literature. This provides new insights into the physicochemical characteristics of the GTA compound and its potential application in the removal of dyes from aqueous systems. Both GTA and GP compounds were evaluated in the form of pellets, so in addition to being effective for the removal of the MB dye, they are easy to handle and recover. In addition, they have good mechanical resistance, which makes them suitable for use in practical applications that improve water quality and protect the environment.

In this study, the practical applicability of the synthesized compounds was focused on wastewater treatment, which could certainly have a significant economic impact by helping industries comply with environmental regulations and avoid penalties for non-compliance. Therefore, the technology developed in this study could be transferred to industries and used in wastewater treatment plants, creating business opportunities and contributing to the development of sustainable solutions in the industrial sector. This would not only benefit the economy but would also promote more environmentally responsible practices.

Although the research shows promising results, several challenges in its implementation were also identified. These challenges include the proper selection of materials and formulation of the compounds, the optimization of the experimental conditions, and the evaluation of the stability and reusability of the compounds over time. Therefore, new possibilities are available for future research to explore the incorporation of other compounds in geopolymeric matrices to further improve their efficiency and carry out applicability studies in real conditions to evaluate the feasibility of their large-scale application. It is also recommended to optimize the methods of synthesis and manufacture of geopolymeric compounds to achieve a more efficient and economical large-scale production, which allows taking advantage of the full potential of these materials in environmental and remediation applications.

In conclusion, this research demonstrates the efficiency of GP and GTA geopolymeric compounds for MB dye removal from aqueous systems and suggests their potential application in wastewater treatment.

## Figures and Tables

**Table 1:** Composition (wt.%) of Kaolin, GP and GTA.

Oxides	Kaolin	GP	GTA
Al_2_O_3_	22.20 (±1.12)	19.62 (±1.07)	11.77 (±0.92)
SiO_2_	37.80 (±2.07)	35.22 (±2.15)	21.13 (±1.84)
K_2_O	1.53 (±0.10)	1.33 (±0.15)	0.82 (±0.01)
CaO	1.92 (±0.13)	1.65 (±0.13)	1.04 (±0.09)
Fe_2_O_3_	0.43 (±0.02)	0.36 (±0.03)	0.22 (±0.01)
TiO_2_	-	-	21.15 (±1.42)
ZnO	-	-	6.72 (±1.05)
Si/Al	2.89 (±0.21)	3.05 (±0.35)	3.05 (±0.34)

**Table 2:** Elemental analysis (wt.%) of kaolin, GP and GTA.

Samples	C	O	Na	Al	Si	K	Ca	S	Ti	Zn
Kaolin	9.12	34.85	1.09	13.34	39.28	1.39	0.92	-	-	-
GP	8.44	34.75	1.73	13.19	38.78	1.72	0.73	0.65	-	-
GTA	5.39	29.17	0.96	4.17	12.72	0.97	0.19	0.37	34.89	11.16

**Table 3:** Point of Zero Charge and Specific Surface Area (m^2^/g) of kaolin, GP and GTA.

Samples	pH_PZC_	SSA (m^2^ g^−1^)
Kaolin (powder)	4.6	36.8
Geopolymer (powder)	6.8	151.3
Geopolymer + ZnTiO_3_/TiO_2_ (powder)	7.2	290.2
Geopolymer (pellet)	6.8	97.8
Geopolymer + ZnTiO_3_/TiO_2_ (pellet)	7.2	187.7

**Table 4:** Isotherm parameters for MB adsorption on GP and GTA pellets at three temperatures.

Isotherm Parameters		293.15 K		298.15 K		303.15 K	
		GP	GTA	GP	GTA	GP	GTA
Langmuir	q_max_ (mg g^−1^)	48.97 (±1.33)	61.96 (±2.52)	53.87 (±1.46)	68.16 (±2.17)	58.76 (±1.60)	74.35 (±3.02)
	K_L_ (L mg^−1^)	0.33 (±0.03)	0.37 (±0.05)	0.40 (±0.04)	0.46 (±0.06)	0.48 (±0.03)	0.54 (±0.04)
	R_L_	0.13	0.12	0.11	0.10	0.09	0.08
	χ^2^	2.01	1.47	2.21	1.62	2.43	1.78
	R^2^	0.99	0.99	0.99	0.99	1.00	0.98
Freundlich	K_F_ (L mg^−1^)	14.94 (±1.79)	20.30 (±2.15)	16.43 (±1.97)	22.33 (±2.37)	17.93 (±2.15)	24.36 (±2.58)
	n	2.81 (±0.36)	2.91 (±0.40)	3.04 (±0.40)	3.20 (±0.44)	3.37 (±0.39)	3.49 (±0.41)
	1/n	0.42	0.41	0.32	0.31	0.30	0.29
	χ^2^	3.67	2.37	3.70	2.61	2.97	2.87
	R^2^	0.94	0.95	0.96	0.95	0.95	0.94

**Table 5:** Thermodynamic parameters of MB adsorption on GP and GTA pellets.

Samples	Temperature (K)	ln k_C_	∆G° (kJ mol^−1^)	∆H° (kJ mol^−1^)	∆S° (kJ mol^−1^ K^−1^)
**GP**	293.15	13.11	−37.98	−27.95	0.22
	298.15	13.29	−39.08		
	303.15	13.49	−40.23		
**GTA**	293.15	13.23	−38.26	−27.97	0.23
	298.15	13.43	−39.43		
	303.15	13.61	−40.52		

**Table 6:** Kinetic parameters of MB adsorption on GP and GTA pellets.

Kinetic Parameters		GP	GTA
Pseudo-first-order	q_max_ (mg g^−1^)	93.63 (±2.24)	94.83 (±2.17)
	k_1_ (L mg^−1^)	0.05 (±4.79 × 10^−3^)	0.06 (±5.30 × 10^−3^)
	χ^2^	2.57	2.26
	R^2^	0.98	0.98
Pseudo-second-order	q_max_ (mg g^−1^)	109.11 (±2.34)	109.21 (±1.74)
	k_2_ (L mg^−1^)	5.57 × 10^−4^ (±5.62 × 10^−5^)	6.43 × 10^−4^ (±4.96 × 10^−5^)
	χ^2^	1.03	0.74
	R^2^	0.99	0.99
Intraparticle diffusion	k_3_ (mg g^−1^ min^−1/2^)	8.80 (±0.11)	8.84 (±0.12)
	*A*	13.14 (±0.27)	12.91 (±0.29)
	R^2^	0.95	0.95
External-film diffusion	D_f_ (m^2^ min^−1^)	5.84 × 10^−12^	6.77 × 10^−12^
	R^2^	0.96	0.94
Internal-pore diffusion	D_p_ (m^2^ min^−1^)	5.10 × 10^−18^	6.00 × 10^−18^
	R^2^	0.93	0.90

**Table 7:** MB adsorption capacity of synthesized geopolymeric materials and of other materials reported in the literature.

Material	q_e_ (mg g^−1^)	References
Py-GP4 porous geopolymer based pyrophyllite	64.10	[31]
Biomass fly ash geopolymer monoliths	20.50	[33]
Metakaolin based geopolymer	7.64	[34]
Metakaolin based geopolymer	43.48	[39]
Diatomaceous earth	77.05	[49]
ZnTiO_3_/TiO_2_-Diatomaceous earth	37.32	[49]
a-TiO_2_/ZnTiO_3_	16.00	[52]
a-TiO_2_	15.00	[52]
Agricultural and industrial waste by-products based geopolymer	0.0449	[78]
TiO_2_/Agricultural and industrial waste by-products based geopolymer	0.0451	[78]
Biomass fly ash based geopolymer spheres	30.10	[79]
Kaolin geopolymer (2009)	25.60	[80]
Metakaolin and rice husk based geopolymer	4.90	[81]
Hierarchical xNGeo particle	115	[82]
Coal FA-geopolymer cube	84.00	[83]
Metakaolin-geopolymer sphere	4.20	[84]
Cu_2_O/TiO_2_ geopolymer nanoparticle	14.76	[85]
Seawater based geopolymer	59.52	[86]
Kaolin	21.41	[87]
Activated lignin-chitosan composite extrudates	36.25	[88]
TiO_2_/montmorillonite-albumin nanocomposite	18.18	[89]
Carboxymethyl cellulose/ZSM-5/ZIF-8	10.49	[90]
ZSM-5 zeolite	105.82	[91]
NaX zeolite	127.13	[92]
Chitosan/clay microspheres	152.20	[93]
Magnetic chitosan/clay beads	82.00	[94]
Activated carbon-clay composite	178.64	[95]
Hydroxysodalite	10.82	[96]
Nonporous silica	91.10	[97]
Natural clay	15.40	[98]
Geopolymer (GP)	48.97	This study
Geopolymer/ZnTiO_3_/TiO_2_ (GTA)	61.96	This study

**Table 8:** Different operating conditions and efficiency for the photocatalytic oxidation of MB by different materials.

Type of Dopant	MB (mg L^−1^)	Type of Light	Reaction Time (min)	Efficiency (%)	Reference
TiO_2_-coated geopolymer spheres	-	UV irradiation	600	93	[99]
Fly ash-based geopolymer	15	UV irradiation	240	92.7	[100]
Geopolymer/TiO_2_-doped-Zeolite	40	UV irradiation	180	99.1	[101]
Geopolymer/Zeolite	40	UV irradiation	180	92.5	[101]
Perlite-based geopolymer	30	UV irradiation	240	97.9	[102]
RMGP	20	UV irradiation	720	94.6	[103]
TBBFS/geopolymer	20	Visible irradiation	180	93.5	[104]
TiO_2_/C	28.5	Visible irradiation	420	70	[105]
TiO_2_/Ce	32	Visible irradiation	180	90	[106]
TiO_2_/Au	12	Visible irradiation	48	92	[107]
TiO_2_/Sb	100	Visible irradiation	60	100	[108]
TiO_2_/C/N	10	Visible irradiation	180	85	[109]
TiO_2_/N/F	5.74	Visible irradiation	140	16	[110]
TiO_2_/Mn/Fe	10	Visible irradiation	150	85	[111]
ZnTiO_3_/PANI/Ag	10	Visible irradiation	25	96	[112]
TiO_2_/N	10	Solar light	120	97	[113]
TiO_2_/F	10	Solar light	120	55	[114]
TiO_2_/La	0.1	UV irradiation	120	85	[115]
TiO_2_/Fe	0.1	UV irradiation	120	75	[115]
ZnTiO_3_/Ag	10	UV irradiation	150	93	[116]
Geopolymer (GP)	20	UV irradiation	150	4.0	This study
Geopolymer/ZnTiO_3_/TiO_2_ (GTA)	20	UV irradiation	150	93	This study

## Data Availability

Data are contained within the article.
